# Where do the treeless tundra areas of northern highlands fit in the global biome system: toward an ecologically natural subdivision of the tundra biome

**DOI:** 10.1002/ece3.1837

**Published:** 2015-12-15

**Authors:** Risto Virtanen, Lauri Oksanen, Tarja Oksanen, Juval Cohen, Bruce C. Forbes, Bernt Johansen, Jukka Käyhkö, Johan Olofsson, Jouni Pulliainen, Hans Tømmervik

**Affiliations:** ^1^ Department of EcologyUniversity of OuluFI‐90014OuluFinland; ^2^ Department of Arctic and Marine BiologyUniversity of Tromsø – The Arctic University of NorwayCampus AltaNO‐9509AltaNorway; ^3^ Section of EcologyDepartment of BiologyUniversity of TurkuFI‐20014TurkuFinland; ^4^ Finnish Meteorological InstitutePL 50300101HelsinkiFinland; ^5^ Arctic CentreUniversity of LaplandP.O. Box 122FI‐96101RovaniemiFinland; ^6^ Northern Research InstituteBox 6434, ForskningsparkenNO‐9294TromsøNorway; ^7^ Department of Geography and GeologyDivision of GeographyUniversity of TurkuFI‐20014TurkuFinland; ^8^ Department of Ecology and Environmental ScienceUmeå UniversitySE‐901 87UmeåSweden; ^9^ The Norwegian Institute for Nature Research (NINA)FramsenteretNO‐9296TromsøNorway

**Keywords:** Alpine, arctic, biome delimitation, ecoregion, mountains, tundra ecosystems, vegetation pattern, winter climate

## Abstract

According to some treatises, arctic and alpine sub‐biomes are ecologically similar, whereas others find them highly dissimilar. Most peculiarly, large areas of northern tundra highlands fall outside of the two recent subdivisions of the tundra biome. We seek an ecologically natural resolution to this long‐standing and far‐reaching problem. We studied broad‐scale patterns in climate and vegetation along the gradient from Siberian tundra via northernmost Fennoscandia to the alpine habitats of European middle‐latitude mountains, as well as explored those patterns within Fennoscandian tundra based on climate–vegetation patterns obtained from a fine‐scale vegetation map. Our analyses reveal that ecologically meaningful January–February snow and thermal conditions differ between different types of tundra. High precipitation and mild winter temperatures prevail on middle‐latitude mountains, low precipitation and usually cold winters prevail on high‐latitude tundra, and Scandinavian mountains show intermediate conditions. Similarly, heath‐like plant communities differ clearly between middle latitude mountains (alpine) and high‐latitude tundra vegetation, including its altitudinal extension on Scandinavian mountains. Conversely, high abundance of snowbeds and large differences in the composition of dwarf shrub heaths distinguish the Scandinavian mountain tundra from its counterparts in Russia and the north Fennoscandian inland. The European tundra areas fall into three ecologically rather homogeneous categories: the arctic tundra, the oroarctic tundra of northern heights and mountains, and the genuinely alpine tundra of middle‐latitude mountains. Attempts to divide the tundra into two sub‐biomes have resulted in major discrepancies and confusions, as the oroarctic areas are included in the arctic tundra in some biogeographic maps and in the alpine tundra in others. Our analyses based on climate and vegetation criteria thus seem to resolve the long‐standing biome delimitation problem, help in consistent characterization of research sites, and create a basis for further biogeographic and ecological research in global tundra environments.

## Introduction

The treeless tundra biome, characterized by low summer temperatures (Köppen 1900; Körner and Paulsen 2004; Körner 2007), consists of arctic and alpine sub‐biomes (Bliss 1956; Billings 1973; Gabriel and Talbot 1984). Unfortunately, there is no consensus about the limits between the two sub‐biomes or about the criteria by which this limit should be determined. In global biome maps, altitudinal extensions of the tundra are routinely regarded as integral parts of the circumpolar arctic; the alpine sub‐biome is restricted to middle‐latitude mountains (Brown and Gibson 1983; Olson et al. 2001; see also Sonesson et al. 1975; Bliss 1981). This broad definition of the arctic tundra is also frequently used in research papers (e.g., Kohler et al. 2006; Hartley et al. 2013) and in climate change studies (Kaplan et al. 2003). In the same spirit, Körner et al. (2011) exclude tundra areas on northern hills and elevated plateaus from the alpine sub‐biome, implying that they rather belong to the arctic. In contrast to Olson et al. (2001), however, Körner et al. (2011) include all “rugged” tundra areas (with local altitudinal differences exceeding 200 m) in the alpine sub‐biome, regardless of latitude or of absolute altitudes. They motivate their focus on topography by pointing out that many specific features of altitudinal zones or belts, which distinguish them from corresponding latitudinal zones, are caused by relative rather than absolute altitudes. Conversely, several authors (e.g., Elvebakk et al. 1999; Moen 1999; Sjörs 1999; Walker et al. 2005) use strictly the polar tree line (tree line at altitude zero) as the southern limit of the arctic, and regard all altitudinal extensions of the tundra as parts of the alpine sub‐biome.

**Figure 1:**
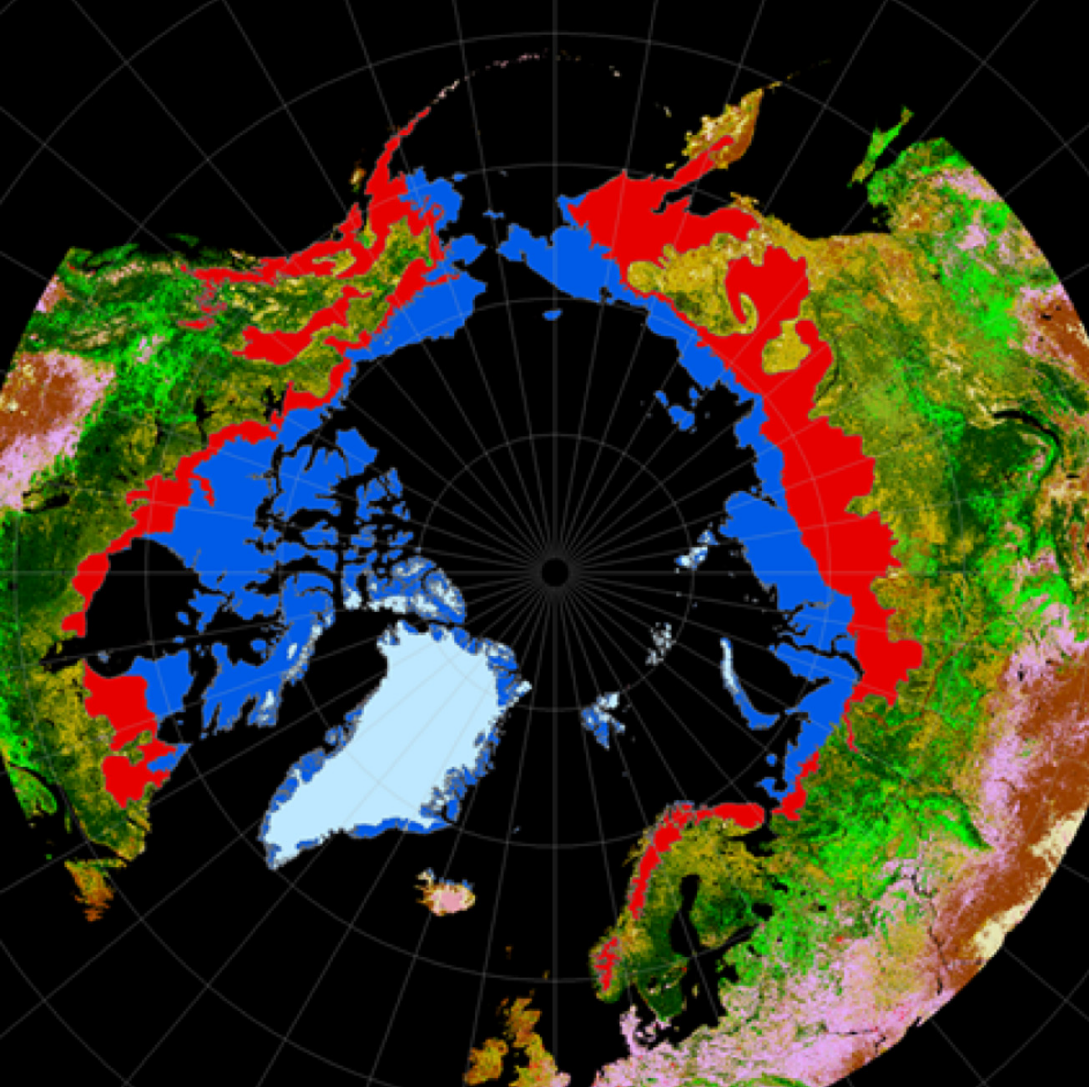
The arctic zone as defined by Walker et al. (2005) (blue) and adjacent areas where treeless tundra is the dominating component of the landscape and which are included in the arctic zone, as defined by Olson et al. (2001) and Kaplan et al. (2003) (red).

The use of different and mutually incompatible criteria has created confusion, both globally and regionally. Globally, the areas of the arctic (5.0 million km^2^) and the alpine (2.9 million km^2^) tundra, as defined by Walker et al. (2005) and Körner et al. (2011), do not sum up to the aggregated area of entire tundra biome (11 million km^2^, Olson et al. 1983). The biogeographic affinities of the three million square kilometers of “missing tundra” remain obscure. Regionally, this discrepancy is reflected by the Fennoscandia tundra, which have been regarded as entirely arctic (Brown and Gibson 1983) or almost entirely alpine (Ahti et al. 1968; Eurola 1974; Moen 1999), except for outer fringes of northern peninsulas (Haapasaari 1988; Walker et al. 2005). The problem is circumpolar: corresponding altitudinal extensions of the arctic tundra cover vast areas in Siberia, Alaska‐Yukon, and on Ungava Peninsula. Moreover, all along the polar tree line, there is a broad transitional zone, where taiga is restricted to lowlands and valleys, while tundra prevails on heights and elevated plateaus (Fig. 1).

In the context of the boreal zones, problems of the same kind were tackled by Ahti et al. (1968). They treated all such altitudinal extensions of boreal zones, which do not substantially rise above their surroundings, as integral parts of the latitudinal zone in question, because of similar bioclimate and vegetation. Conversely, they named altitudinal extensions of arctic zones in north‐western Europe to as oroarctic, which in their terminology is synonymous with alpine.

The greatest differences between arctic and alpine bioclimate areas have been proposed to concern winter temperatures and snow precipitation (Oksanen and Virtanen 1995). The cold arctic winters with little precipitation differ radically from the mild and snowy alpine ones (Walter and Lieth 1960) and this contrast has profound ecological ramifications. Due to the scanty snow cover, arctic ground temperatures are close to the frigid monthly average air temperatures (Dingman et al. 1980; Coulson et al. 1995; Hinkel and Hurd 2006). Consequently, permafrost is widespread (Brown et al. 1997; Romanovski 2011), and the topsoil remains frozen after the snowmelt. Arctic plants thus have to cope with spring drought, which is especially stressful for evergreens (Kullman 1989). On high mountains, permafrost is restricted to windblown ridges and summits (Brown et al. 1997; Harris et al. 2009). Elsewhere, mild air temperatures and thick insulating snow ensure that soil temperatures are close to or above freezing point throughout the winter (Ellenberg 1978; Neuner et al. 1998; Körner et al. 2003). There are also many other differences between arctic and alpine bioclimates that concern the seasonal pattern of moisture, nutrient mineralization rates, magnitude of diurnal temperature variation, intensity of solar radiation, and wind velocity (Bliss 1956; Walter 1968; Billings 1973; Eurola 1974; Nagy and Grabherr 2009).

Today, adequate vegetation descriptions and climate data exist for several low arctic and low alpine tundra areas of western Eurasia (western Siberia, European Russia, Fennoscandia, the Alps, and the Pyrenees). In this region, all tundra areas derive their species from the same pool, the Pleistocene tundra of Central and Eastern Europe (Birks 2008; Eidesen et al. 2013). We can thus assume that contrasts in species composition reflect regional differences in ecological conditions and are little, if at all, influenced by history or by dispersal barriers. In studies covering larger and floristically more heterogeneous areas (e.g., Walker et al. 2005), plants need to be pooled to functional plant types in order to ensure that impacts of dispersal barriers do not influence results. These functional groups are inevitably ecologically heterogeneous (van Bodegom et al. 2012; Wullschleger et al. 2014). Hence, their abundance relationships contain less information than the distribution and abundance relationships of individual species. To ensure that detected patterns reflect differences in bioclimate rather than in bedrock geology, we will focus on the vegetation of such well‐drained sites, to be referred to as tundra heaths, which are neither influenced by running water nor by exceptionally nutrient‐rich bedrock. Moreover, we will tackle the complexity of the tundra vegetation, caused by uneven snow distribution, by defining the regional tundra vegetation as the characteristic sequence of heath communities from bare‐blown ridges to snow accumulation sites. The attributes of the regional tundra vegetation thus consist of the composition of individual heath communities and of their abundance relationships along local topographic gradients (Oksanen and Virtanen 1995).

Our goal is to settle the controversies outlined above by studying patterns in bioclimate and in tundra heath vegetation within all those parts of western Eurasia, where data are available. In this effort, we will use (1) climate data from weather stations, (2) satellite‐based temperature data, (3) vegetation data, (4) and satellite‐based data on abundance relationships between different heath community types. Using the above‐described approach, we hope to arrive to an ecologically natural subdivision of the tundra biome, which will help ecologists to upscale results of local experimental studies to ecologically comparable parts of the tundra biome.

## Material and Methods

### Broad‐scale comparison of tundra sites from the Pyrenees and the Alps to the Siberian tundra

#### Study sites

**Figure 2:**
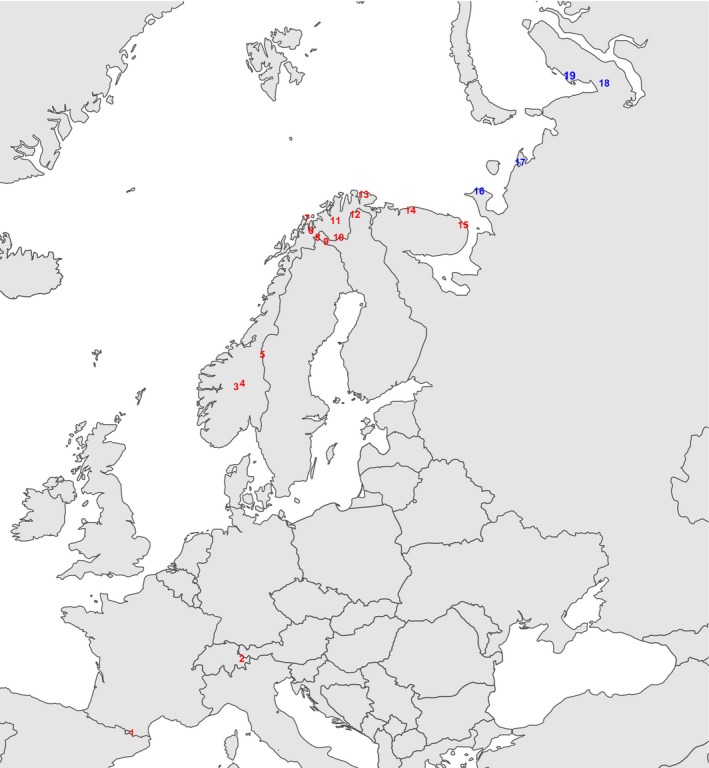
The locations of the 19 sites from which sample plot data were used in the analysis of tundra vegetation patterns. The Pyrenees (1), The Alps (2), southern Scandes (3–5), northern Scandes (6–9), interior Finnmark and northern Finnish Lapland (10,11), coastal Finnmark (13), Kola Peninsula (14,15), Kanin Peninsula (16), Pechora Peninsula (17), and Yamal Peninsula (18,19). The arctic sites (Walker et al. 2005) are shown in blue, other treeless tundra sites are shown in red.

In order to maximize the homogeneity of our study sites in aspects other than their position along the axis from arctic to alpine areas, we will focus on tundra areas lying at or above/north of timber line (the mean temperature of the warmest month c. +8–12°C). These include low arctic/alpine areas that are clearly treeless, hemiarctic/orohemiarctic areas that have features of both boreal forest and treeless tundra (Ahti et al. 1968), usually so that treeless tundra prevails but patches of forest are present in microclimatically favorable sites (lesotundra sensu Norin 1961; Crawford 2013). These tundra areas form a fairly continuous arch from Siberia to the Pyrenees, though with a gap between 59°N (the southernmost Scandes) and 47°N (the northernmost Alps). Based primarily on the availability of appropriate vegetation data sources, we chose 19 study sites representing these tundra areas (Fig. 2).

#### Climate data

We first explored available gridded fine‐scale climate data from global databases (such as WorldClim; Hijmans et al. 2005) for the study sites, but found these inadequate in coverage or potentially highly biased (especially for mountainous areas). Therefore, our main sources of climate data originate from weather stations close to the tree line and selected study sites. A general problem was very limited availability of suitable climate stations. A very close matching with the vegetation data was thus unfeasible. However, we succeeded in retrieving comparable data on annual mean temperature, annual precipitation, July mean temperature, June–August precipitation, average temperatures, and snow depth in January–February for 38 stations, from the same regions where our vegetation data were obtained, except for the Pyrenees, where no appropriately located weather stations could be found (Appendix S1). As the extent of soil frost depends on temperatures and the depth of the insulating snow cover during the coldest months, we especially focused on average temperatures and snow depth in January–February. When possible, we excluded stations in narrow valleys and highly wind‐exposed sites, as these exhibited anomalous microclimate and snow depth. However, we had to relax the latter criterion in the context of coasts of northernmost Norway and the Alps, because in these areas, all complete climate stations representing the low arctic/low alpine zones were located in exceptionally wind‐exposed sites (lighthouses, ridges, summits). We also checked whether there were other biases in the locations of climate stations. This was the case on the northern Scandes, where all appropriately located stations were found on east‐facing slopes, with relatively cold and dry climate. These biases will be discussed when interpreting the data.

In our analyses of climate patterns, we grouped the 19 study sites into the following nine regional units: Yamal Peninsula, Pechora (coast between Kanin Peninsula and the Urals), Kanin Peninsula, Kola Peninsula, coastal Finnmark, interior Finnmark, northern Scandes, southern Scandes, and the Alps. For each part, we computed means and standard deviations of station means for temperature and snow depth in January–February, using those several years' periods for which data were available (Appendix S1).

#### Plant community data

The data sets on tundra heath vegetation from Siberia to the outer islands of NW Norway have been systematically sampled with the same method everywhere (see Oksanen and Virtanen 1995). Data from southern Norway, Alps, and Pyrenees were obtained from classical monographs and are thus based on subjective sampling, but also in these data sets, the entire gradient from ridges to depressions is represented (see Appendix S2 for data sources). For the tundra areas of Russia and northern Fennoscandia, we used vegetation data archives assembled for previous vegetation studies (Oksanen and Virtanen 1995; Virtanen et al. 1999, 2006) and some unpublished data (B.C. Forbes, H. Tømmervik). For the southern Fennoscandian tundra and for the mountains of central and southern Europe, we used data obtained from monographies covering comparable areas (Nordhagen 1943; Dahl 1957; Virtanen et al. 2003; Braun‐Blanquet 1948; Vetterli 1982). In the numerical vegetation ordination analyses, 30–80 plots from each study site were included. In total, the data set used for the vegetation ordination analyses contains 1200 sample plots with cover estimations on vascular plants, bryophytes, and lichens. The summaries of the vegetation categories included in the analysis are given in Appendix S2.

### Remote sensing analysis of Fennoscandian tundra vegetation patterns and winter climate

For western Fennoscandia, we also studied abundance relationships between different tundra heath types using a vegetation map of B. Johansen (unpublished data, see also Johansen 2009; Johansen et al. 2009; Cohen et al. 2013; Johansen et al. 2012; Johansen and Karlsen 2005, 2008), based on 39 Landsat TM/ETM+ images. The spatial resolution of the map is 100 m. The vegetation map was differentiated into 21 map units; eight of these were different tundra heath types, which correspond roughly to the community groups of Oksanen and Virtanen (1995) and were named accordingly.

To compare characteristics of heath type distributions and climate conditions in the Fennoscandian tundra, we selected twelve 25 × 25 km quadrats from the ECMWF database encompassing Finnmarksvidda, Norway, Enontekiö, Finland, and northernmost Swedish Lapland, two from basins within the mountain chain and four from its southern and eastern flanks. The selection criterion was that tundra prevails, but >90% of the land lies <200 m above the tree line (Appendix S3). Thereafter, we performed ordination and cluster analysis of these twelve quadrats, with relative abundances of these eight tundra heath types as input variables (see below for more details).

January–February temperatures for the 12 25 × 25‐km quadrats in Fennoscandia were obtained from the satellite‐based ECMWF database (the ERA‐Interim Archive of European Centre for Medium Range Weather Forecast). The product provided gridded surface temperatures with a spatial resolution of 1.5 degrees. In the grids, temperatures are interpolated globally, which allowed the computation of temperature estimates for all 12 quadrats. Within each quadrat, several mean monthly January–February temperature values were sampled and these were averaged for 1982–2010.

### Numerical analyses of plant community and vegetation data

We explored the similarities and differences in the composition of 1200 plant community sample plots from different tundra sites by means of nonmetric multidimensional scaling analysis (NMDS; Minchin 1987). We first transformed original species' cover classes to % cover scale. This was made separately for each data set following Oksanen (1976) for data sets having Hult–Sernander cover class or its extended form. In this transformation, cover classes 1–10 got % cover estimates as follows: 1 = 0.125%, 2 = 0.25%, 3 = 0.5%, 4 = 1.1%, 5 = 2.2%, 6 = 4.4% 7 = 8.9%, 8 = 17.9%, 9 = 35.6%, and 10 = 71.2%; the transformation of Hult–Sernander +‐5 scale to % scale + = 0.25%, 1 = 4.4%, 2 = 8.8%, 3 = 17.8%, 4 = 35.6%, 5 = 71.2%; the transformation of Braun–Blanquet scale to % scale r = 0.1%, + = 0.25%, 1 = 2%, 2 = 11.2%, 3 = 35.4%, 4 = 61.2%, and 5 = 86.6%; and the transformation of Domin scale (Dahl 1957) to % scale + = 0.1%, 1 = 0.1%, 2 = 0.25%, 3 = 2%, 4 = 6.3%, 5 = 15.8%, 6 = 28.7%, 7 = 40.6%, 8 = 61.2%, 9 = 82.2%, 10 = 94.9%. These transformations to % scale thus maintain information on species relative abundances and give weight to dominant species (van der Maarel, 1979). The NMDS analysis was run using the metaMDS function of vegan (Oksanen et al. 2015) for transformed **%**‐cover class data (Bray‐Curtis dissimilarity metric). The function used Wisconsin double standardization and square‐root transformation. The same NMDS analysis methods were used for the mapped Fennoscandian tundra community type data. The clustering analyses of mapped tundra vegetation were run using agglomerative hclust R function with ‘complete linkage’ option (R Core Team, 2013).

## Results

### Patterns in climate

**Figure 3:**
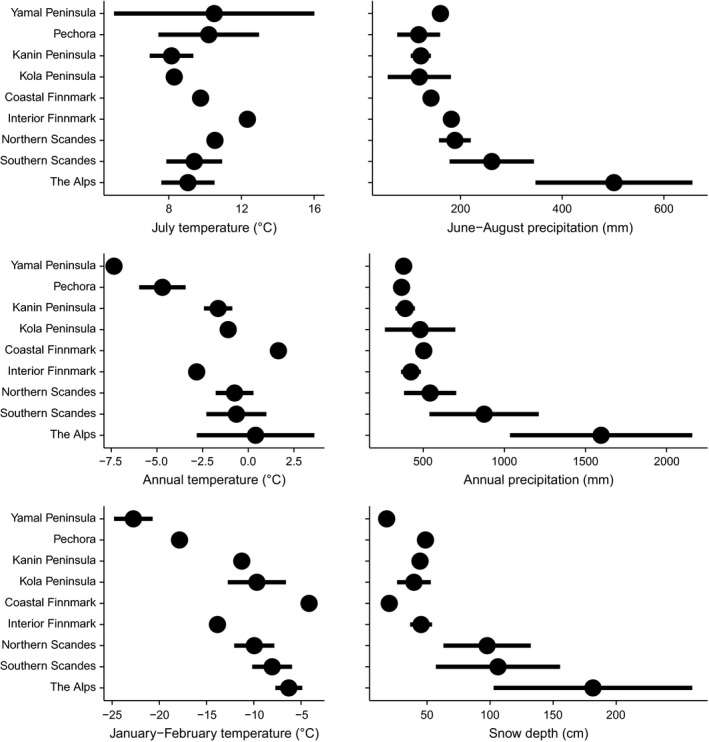
Mean July, annual, and January–February temperatures and mean June‐August, annual precipitation and mean snow depth for nine arctic and alpine regions derived from data available from 38 climate stations (for the climate stations included, see Appendix S1).

There is no clear trend pattern in July mean temperature from the Siberian tundra to the Alps, and the temperatures are mostly +8–10°C (Fig. 3). This matches with our site selection criterion to only include relatively similar tundra areas in terms of summer thermo‐climate. Unavoidably, some within‐ and among‐site variation in temperature patterns exists, due to the scarcity of climate stations in the tundra sites. For instance, in Yamal Peninsula, the widely scattered observations result from necessity to include station records from a colder (Marresale) and warmer site (Salekhard). Differing from summer temperature, clearer trend patterns can be seen in annual temperature, as well as annual and summer precipitation (Fig. 3). These patterns primarily highlight the contrast between middle‐latitude mountains with high precipitation and mild thermal conditions, arctic tundra areas with low precipitation and cold thermal conditions.

Winter temperatures display a pronounced gradient from western Siberia to the coasts of Finnmark, Norway, with January–February average ranging from <−20°C to about −5°C, respectively (Fig. 3). The inland plateau of Finnmarksvidda is characterized by frigid winter temperatures of about −14°C, while on the Scandes, we again encounter milder winter conditions. There is a gradient of increasing winter temperatures from northern Scandes to southern Scandes and to the Alps, but due to the biases in station locations, the representativeness of this trend is uncertain. An alternative way to interpret the data from areas with rugged topography is that in mountainous parts of the European tundra, the mean January–February temperature lies between −5 and −12°C; ridges are the mildest sites during winter while east‐facing valleys exhibit the lowest temperatures.

Mean January–February snow depth is consistently <50 cm along the entire gradient from western Siberia to Finnmark, Norway (Fig. 3), but the snow depths along the northern coast of Norway may be grossly underestimated, due to the locations of climate stations. On the Scandes, mean January–February snow depth is about 100 cm and there seems to be a trend of increasing snow depth from north to south (Fig. 3). However, stations on the east slopes have similar values throughout the Scandes, that is, the east slope bias in the northern stations can account for or contribute to this trend. However, being in line with the increasing north–south gradient of snow depth in Fennoscandia, snow depths in the Alps are about 180 cm, and these values are probably deflated, due to the positions of weather stations on ridges.

### Similarities and dissimilarities between arctic and alpine plant communities

**Figure 4:**
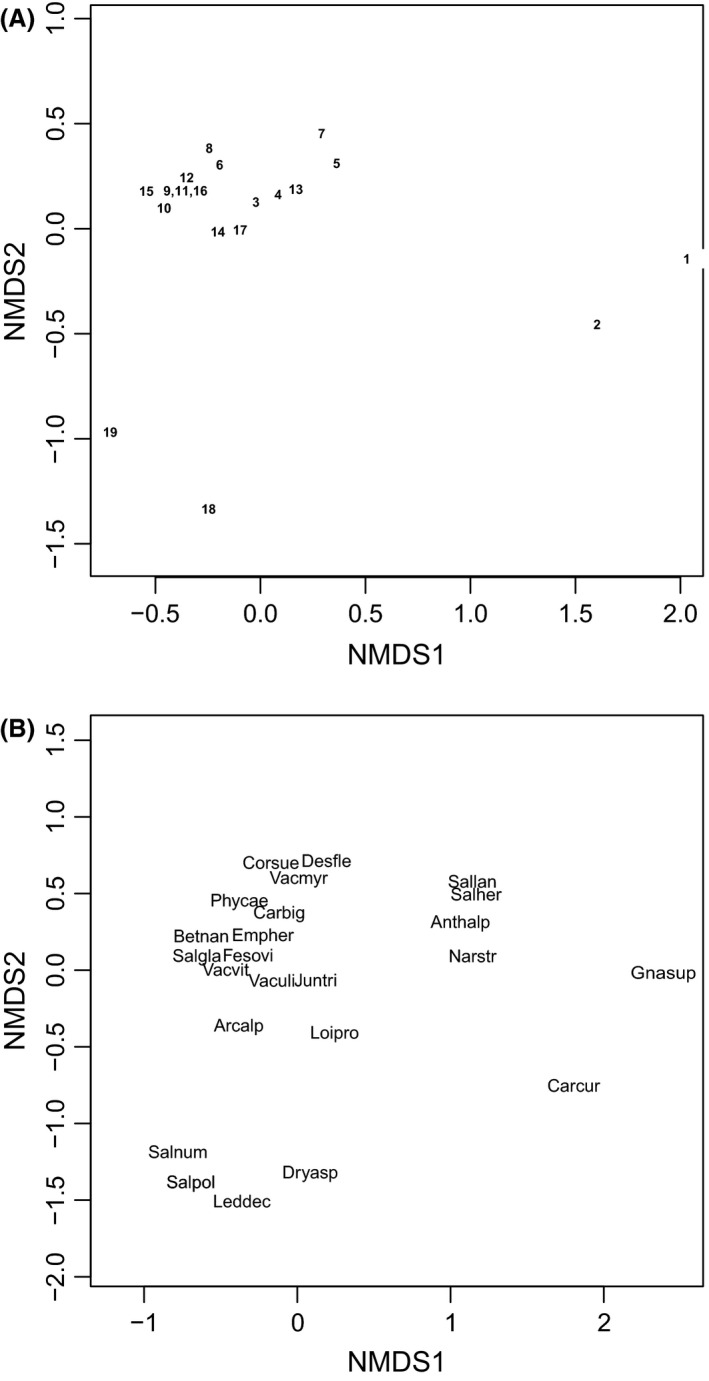
(A) Ordination of tundra plant communities from European middle‐latitude mountains to western Siberia. The numbers show the centroids of the 19 tundra sites (see Fig. 2). (B) The ordination scores of the most abundant and/or characteristic vascular plant species in the plant community data. Anthalp = *Anthoxanthum alpinum*, Arcalp = *Arctostaphylos alpina*, Betnan = *Betula nana*, Carbig = *Carex bigelowii*, Carcur = *Carex curvula*, Corsue = *Cornus suecica*, Desfle = *Deschampsia flexuosa*, Dryassp = *Dryas octopetala*, Empher = *Empetrum nigrum* (ssp. *hermaphroditum*), Fesovi = *Festuca ovina*, Gnasup = *Gnaphalium supinum*, Juntri = *Juncus trifidus*, Leddec = *Ledum decumbens*, Loipro = *Loiseleuria procumbens*, Narstr = *Nardus stricta*, Phycae = *Phyllodoce caerulea*, Vacmyr = *Vaccinium myrtillus*, Salher = *Salix herbacea*, Salgla = *Salix glauca*, Sallan = *Salix lanata*, Salnum = *Salix nummularia*, Salpol = *Salix polaris*, Vaculi = *Vaccinium uliginosum*, and Vacvit = *Vaccinium vitis‐idaea*.

The pattern of mean site‐scores of the nonmetric multidimensional scaling shows that North European tundra – from southern Scandes to easternmost European Russia – differs clearly from the majority of the alpine tundra communities of Central Europe and from the more continental tundra of western Siberia (Fig. 4A). The ordination scores of the most abundant species (Fig. 4B) show that the North European tundra communities are characterized by prevalence of dwarf shrubs (e.g., the dwarf birch (*Betula nana*), the northern crowberry (*Empetrum nigrum* ssp. *hermaphroditum*) with some graminoids and herbs (e.g., the wavy hair grass (*Deschampsia flexuosa*). The Central European alpine vegetation is characterized by graminoids (e.g., *Carex curvula*) and other chionophilous plants (e.g., the dwarf cudweed (*Gnaphalium supinum*). In the West Siberian tundra communities, we find erect shrubs (e.g., the Labrador tea (*Rhododendron tomentosum* ssp. *decumbens*, also known as *Ledum decumbens*) and such dwarf shrubs, which in northern Europe are confined to areas with exceptionally base‐rich bedrock (e.g., the mountain avens [*Dryas* sp.], the polar willow [*Salix polaris*]).

**Figure 5:**
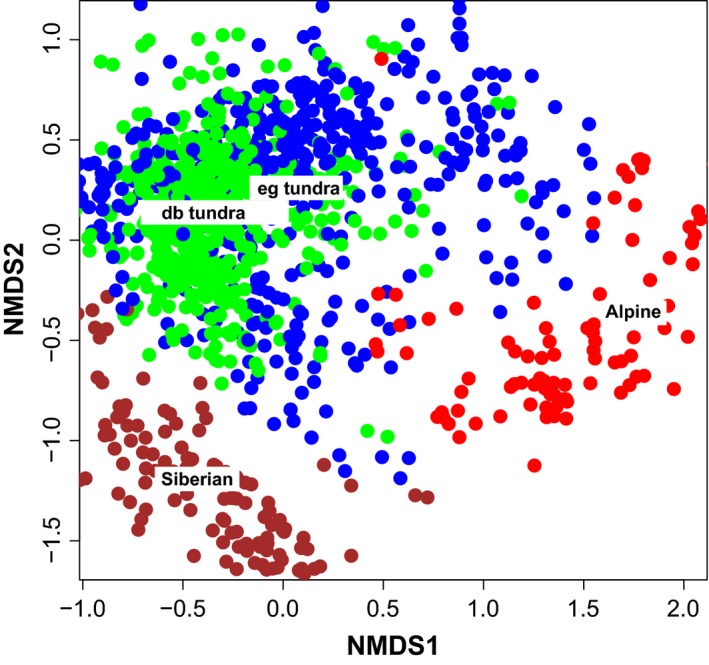
Ordination of hemi to low arctic/low alpine tundra plant communities from European middle‐latitude mountains to western Siberia showing pattern of plots representing four sub‐biome categories (color and the numbers of sites included in Fig. 2): alpine the Alps and the Pyrenees (red; 1,2), Siberian tundra (brown; 18,19), northern European dwarf birch tundra sub‐biome (db tundra shown with green dots; 9,10,11,12,14,15,16,17), and northern European ericoid–graminoid tundra (eg tundra shown with blue dots; 3,4,5,6,7,8,13). Some aberrant sample plots score outside of the ordination space.

In spite of the homogeneity of the North European tundra at the community level, the locations of sample plot centroids (Fig. 4A) and the distribution of individual sample plots (Fig. 5) indicate a moderate degree of differentiation. The tundra of northern Russia, interior Finnmark, and northern Finland concentrate to the same part of the ordination space with deciduous, drought‐hardy shrubs and dwarf shrubs, such as the dwarf birch, the alpine bearberry (*Arctostaphylos alpina*), and the bog bilberry (*Vaccinium ulignosum*), whereas the mean site‐scores and sample plots from the North Norwegian coast and southern Scandes are slightly separated in the ordination space and characterized by evergreen dwarf shrubs on bare‐blown ridges (mainly the alpine azalea, *Loiseleuria procumbens* and the northern crowberry) while the semievergreen bilberry (*Vaccinium myrtillus*), the herbaceous dwarf cornel (*Cornus suecica*), and several species of graminoids abound in sites with deeper snow. The former kind of tundra is labeled as dwarf birch tundra in Figure 5; the latter is labeled as ericoid–graminoid tundra.

Sample plots representing the ericoid–graminoid tundra are scattered over a wide area in the ordination, indicating pronounced heterogeneity of the vegetation, as also emphasized in the primary sources (Appendix S2). Some sample plots intermingle with data points from the dwarf birch tundra and others with data points from northern Fennoscandian coasts. The overall community pattern of the ordination is compatible with the pattern of increasing snow depth and increasing winter temperatures from Siberian tundra to the Alps and mountains and with the similarity of winter climate in the European part of the Russian tundra and in the inland of northernmost Fennoscandia (Fig. 3).

### Tundra vegetation patterns and winter climate in western Fennoscandia

**Figure 6:**
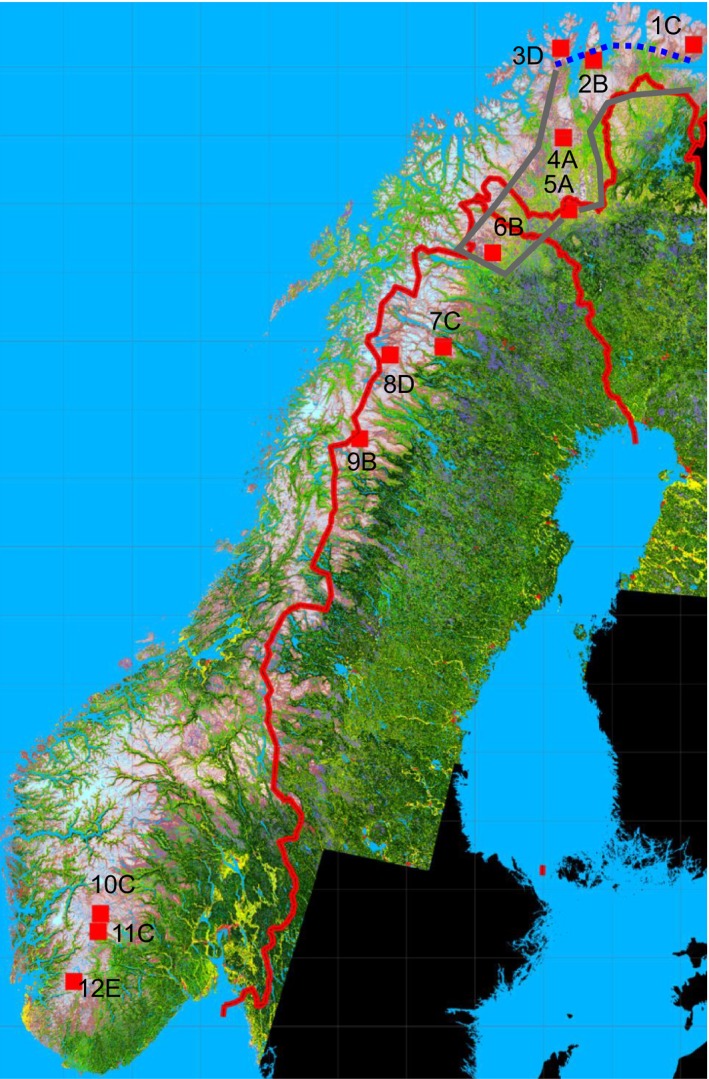
Distribution of tundra (purple‐white), mountain birch forest (bright green), and coniferous forest (dark green) in northern and western Fennoscandia excluding Russian territory. The deep purple represents dwarf birch heaths. The red squares and numbers refer to the locations of the twelve 25 × 25 km quadrats for which habitat distribution was computed from classified satellite images and winter temperatures were assessed by the satellite‐based method. The capital letters refer to the clusters to which the areas were divided on the basis of their habitat distributions. The thick gray line limits the area where the evidence presented by us indicates that the tundra can be regarded as arctic without the oro‐prefix. The dashed blue line denotes the approximate limit of the maximally oceanic sector of the Fennoscandian arctic, which, with respect to ecological conditions and vegetation, is more similar to the Scandinavian ericoid‐graminoid than to the East European dwarf birch tundra, which prevails in the inland. Simplified from the original vegetation map of B. Johansen (unpublished).

**Figure 7:**
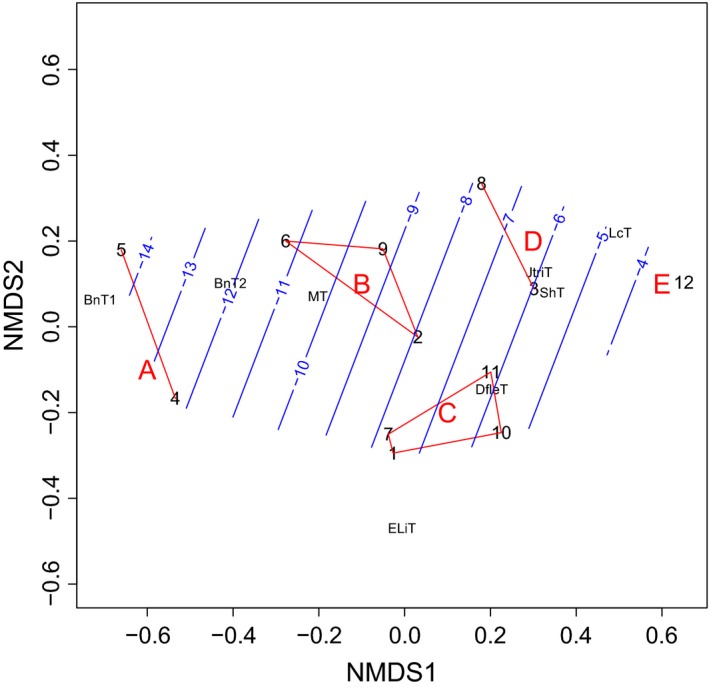
Ordination of the Fennoscandian tundra areas used in the satellite‐based climate and vegetation studies on the basis of abundance relationships between different types of heath vegetation, including snowbeds. Red lines combine five clusters (A–E) based on a complete linkage cluster analysis. The fitted trend surface indicates the winter temperature (January–February °C) gradient (blue lines). The black numbers refer to the twelve 25 × 25 km quadrats shown in Figure 6. The vegetation types: ShT = *Salix herbacea* type = dwarf willow snowbed; DfleT = *Deschampsia flexuosa* type = moist, grassy snowbed; JtriT = *Juncus trifidus* type = grassy, dry snowbed; MT = *Myrtillus* type = bilberry‐purple heather heath, BnT = *Betula nana* type = dwarf birch heath (BnT1: with continuous lichen cover, BnT2: with scanty lichen cover or moss‐dominated bottom layer); ELiT = *Empetrum* lichens type = ridge heath with continuous vegetation; LcT = *Luzula arcuata* ssp. *confusa* type = ridge heath with discontinuous vegetation.

The ordination of the twelve 25‐by‐25‐km quadrats (Fig. 6) on the basis of relative proportions of the eight heath community types shows a gradient that is closely related to mean January–February temperature (Fig. 7, see also Table 1). The cluster analysis divided the twelve quadrates into four clusters and one outlier, arranged primarily along a gradient of increasing abundance of snowbed communities (Salix herbacea type, Deschampsia flexuosa type, and Juncus trifidus type), and decreasing abundance of dwarf birch heaths (Betula nana types, Fig. 7).

**Table 1:** Percentages of different heath types out of the total heath area in the 12 analyzed quadrats of 25 × 25 km. LcT = Luzula confusa type (extreme windbarren) ELiT = Empetrum lichens type (ridge heath with scanty snow cover), BnT1 =  Betula nana – lichens type (dwarf birch heath with copious lichen cover), BnT2 =  Betula nana type (dwarf birch heath or scrub with scanty or moderate lichen cover), MT = Myrtillus type (bilberry – purple heather heath), JtriT = Juncus trifidus type (dry, graminoid‐rich snowbed), DfleT = Deschampsia flexuosa type (moist, graminoid rich snowbed), ShT = Salix herbacea type (late‐melting snowbed with mosses and dwarf willows) The letters in parentheses refer to the clusters to which each quadrat was assigned. Mean January–February temperatures (^o^C) given in the rightmost column

Quadrat	LcT	ELiT	BnT1	BnT2	MT	JtriT	DfleT	ShT	Temp
4(A)	4	15	16	33	23	2	3	5	−12
5(A)	6	2	16	32	35	1	3	6	−14
2(B)	7	13	0	17	32	5	10	16	−7
6(B)	1	2	12	20	38	4	12	11	−13
9(B)	9	6	0	26	34	6	2	16	−9
1(C)	9	20	0	10	31	1	8	22	−6
7(C)	1	19	0	19	25	3	8	24	−12
10(C)	3	20	7	6	21	7	17	19	−5
11(C)	9	12	7	11	20	8	13	20	−5
3(D)	16	6	0	10	18	5	8	37	−5
8(D)	11	3	0	13	27	6	3	37	−9
12(E)	34	3	4	4	5	8	10	29	−3

**Figure 8:**
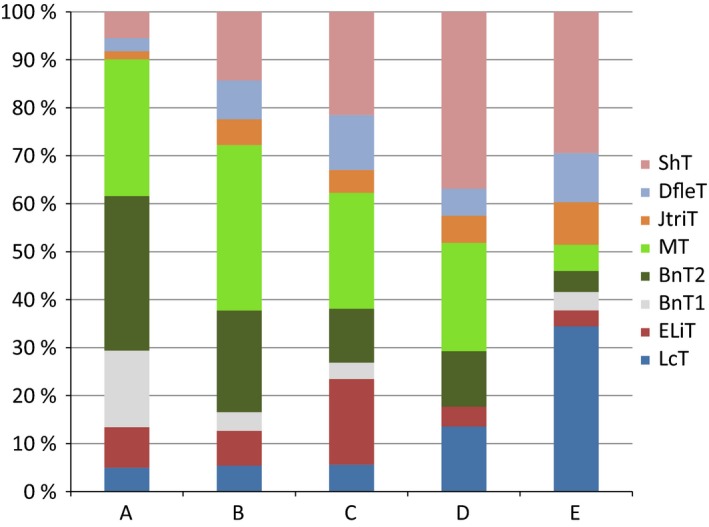
Abundance relationships between different tundra heath types in the five clusters (see Fig. 7) obtained on the basis of these abundance relationships. ShT = *Salix herbacea* type = dwarf willow snowbed; DfleT = *Deschapsia flexuosa* type = moist, grassy snowbed; JtriT = *Juncus trifidus* type = dry, grassy snowbed; MT = *Myrtillus* type = bilberry‐purple heather heath, BnT = *Betula nana* type = dwarf birch heath (1: with continuous lichen cover, 2: with scanty lichen cover or moss‐dominated bottom layer); ELiT = *Empetrum* lichens type = ridge heath with continuous vegetation; LcT = *Luzula arcuata* ssp. *confusa* type = ridge heath with discontinuous vegetation. The locations of quadrats belonging to each cluster are provided in the map in Figure 6.

**Figure 9:**
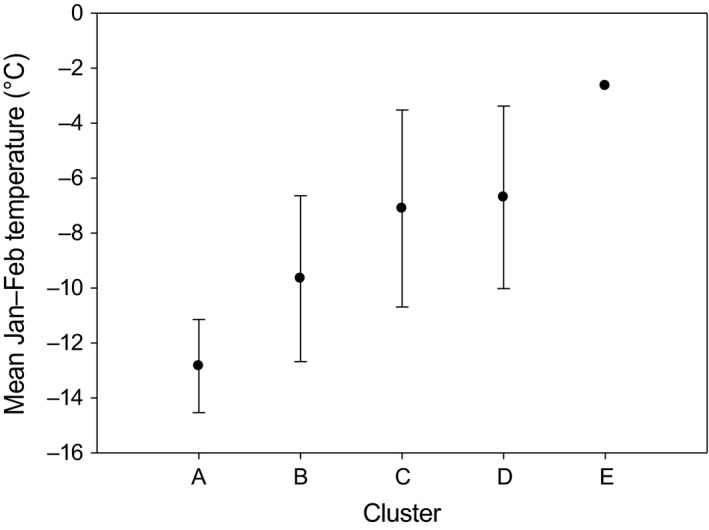
Means and standard deviations of average January–February temperatures (^o^C) of the five quadrat clusters (Figs 6 and 7), interpolated using 25 × 25 km resolution for 1982–2010 data.

Cluster A, representing the northern Fennoscandian inland (Finnmarksvidda, Norway, and its extension to north‐eastern Enontekiö, Finnish Lapland), is distinguished from the rest by the overwhelming prevalence of dwarf birch heaths (Fig. 8) and by cold winters (Fig. 9). Snowbeds cover only about 10% of the terrain and chionophobous heaths are almost equally uncommon, reflecting a calm and cold winter climate. Cluster B is intermediate between Cluster A and the rest with respect to winter temperatures and vegetation patterns. It is represented by three quadrats on the leeward sides of high mountains. Clusters C and D embrace six quadrats with wide latitudinal range from southern Scandes to northern peninsulas. These quadrats are characterized by mild winters (January–February average about −7°C). Snowbeds abound, covering ca. 40–50% of the landscape. Also bare‐blown heaths are common, covering about 20% of the landscape. Sites with intermediate snow cover are primarily occupied by heaths of bilberry and purple heather type. Of these two, Cluster C is more continental, with lower abundance of snowbeds and with lichen‐rich dwarf birch heaths present. The quadrat (E) from Sirdals‐heiane in southernmost Norway forms an outlier, characterized by very mild winters and high abundance of both snowbeds and chionophobous heaths (Figs 8 and 9). Heaths characterized by intermediate snow condition cover only about 13% of the landscape (Table 1).

## Discussion

Our results conform to some extent with the delimitations of the tundra sub‐biome proposed by Olson et al. (2001), who regard the altitudinal extensions of the tundra on northern mountains and heights as integral parts of the circumpolar arctic, but other aspects of our results are rather consistent with the more restrictive concept of the arctic tundra, proposed by Walker et al. (2005). Also the arguments of Körner et al. (2011), who emphasize the importance of relative altitudes, obtain some support. These contradictory aspects of our results motivated us to challenge the premise that the most natural subdivision of the tundra is to arctic and alpine sub‐biomes (Billings 1973; Gabriel and Talbot 1984). We addressed this question by employing multi‐criterion macro‐scale analyses on similarities and dissimilarities of ecological conditions and vegetation, which should ensure the robustness of our conclusions. Our approach and analyses provide several lines of evidence to advance a new biome level patterning that will be useful for placing ecological research sites in correct biogeographic context.

The main dilemma in our results is the mismatch between patterns in winter climate and in the composition of individual heath communities. The winter climate of the easternmost part of the European tundra differs only marginally from the West Siberian winter climate, and there is a gradient of increasing winter temperatures and increasing snow depth from western Siberia to southern Norway, where winters are almost as mild and snowy as on the Alps. Nevertheless, the entire North European tundra harbors comparatively homogeneous heath communities, distinct from both the truly alpine heath vegetation of Central and South European mountains and from the west Siberian tundra vegetation. Differences in Quaternary geology and drier climate where nutrients are not leached from the top soil offer a plausible explanation for the sharp contrast between the tundra heaths of easternmost Europe and westernmost Siberia (Virtanen et al. 1999). The equally sharp contrast between the tundra heath vegetation of the Scandes and the Central and South European mountains is more enigmatic.

Historical reasons are unlikely to account for the contrast between plant communities of these middle‐latitude mountains and Fennoscandia. Our plant community data included 594 taxa with 285 vascular, 164 bryophyte, and 147 lichen taxa. Especially the spore‐dispersing bryophytes and lichens with high dispersal ability (Lenoir et al. 2012) can be regarded as sensitive indicators of climatic conditions. We also note that the distributions of the quantitatively dominating vascular plants are very wide, indicating that they could flourish in any part of western Eurasia with ecologically suitable conditions. Many species typical for the North European tundra are also present as rarities on European middle‐latitude mountains (Coker and Coker 1973) or occur on the foothills as bog plants (de Groot et al. 1997; Jacquemart 1998), indicating that their rarity or absence from alpine tundra heaths has ecological reasons. Also the majority of typical alpine vascular plants growing on nutrient‐poor substrates are present in northern Europe. The few exceptions are either especially sensitive to the drought stress generated by frozen soils (e.g., the rusty leaved azalea (*Rhododendron ferrugineum*, see Neuner et al. 1998) or are graminoids (e.g., the sedge *Carex curvula*), which are, as a group, much less abundant on the North European tundra than on Central European mountains. Notice also that the one habitat category not influenced by the higher snow precipitation of the Central European mountains – the bare‐blown, exposed ridges – is dominated by the northern crowberry and the alpine azalea both on European middle‐latitude mountains and on the Scandes, suggesting that similar ecological conditions would have resulted in similar vegetation in other habitats, too.

Thresholds and other nonlinear effects in the relationship between climate and ecological conditions could account for the apparent discrepancy between the seemingly modest contrasts between the winter climates of the Alps and the Scandes and the pronounced differences in heath communities. A possible feedback loop exists between soil processes and graminoid abundance: warmer soils enhance decomposition rate, which favor graminoids, while the higher abundance of graminoids leads to production of easily decomposed litter, which further accelerates decomposition (Wookey et al. 2009). The processes favoring graminoids probably have opposite effects on bryophytes and lichens, which are much more prevalent on the North European tundra than on middle‐latitude mountains. This argument is supported by the high abundance of graminoids and low cover of mosses and lichens in those North European tundra communities, which have thick snow cover and are underlain by nutrient‐rich bedrock.

Also summer herbivory favors graminoids, enhancing the loop described above (Olofsson et al. 2004), and the composition of the herbivore guild, which influences the timing of maximally intense herbivore–plant interactions, differs between Scandes and middle‐latitude mountains. The vertebrate herbivore guild of the Scandes is entirely arctic, consisting of lemmings, voles, reindeer, and ptarmigans. Browsing by reindeer is especially damaging for tall deciduous shrubs, thus favoring prostrate ericoids at normal grazing intensities (Olofsson et al. 2001, 2009; Tømmervik et al. 2004; Bråthen et al. 2007). Only locally is summer grazing by reindeer intense enough to change scrublands and heaths to grasslands (Olofsson et al. 2001, 2004). Herbivory by lemmings and voles, which strongly contributes to the structuring of the Fennoscandian tundra vegetation (Virtanen 2000; Ravolainen et al., 2013; Olofsson et al. 2012, 2014), occurs primarily in winter. On the middle‐latitude mountains, windy conditions favor harvesting pikas (*Ochotona* spp.), while unfrozen soils provide a favorable environment for the hibernating marmots, which thus can exert strong summer grazing pressure on alpine vegetation (Huntly 1987; Oksanen and Oksanen 1989; Allainé and Yoccoz 2003; Hall and Lamont 2003; McIntire and Hik 2005). On the Central and South European mountains, these native herbivores have long ago been decimated or driven to extinction, but domestic herbivores have taken their role, maintaining intense summer grazing pressure (Ellenberg 1978).

The vegetation data imply that in any either–or decision, the Fennoscandian highland tundra, whether flat or rugged enough to be included in the alpine sub‐biome defined by Körner et al. (2011), has greater affinities to the arctic than to the alpine tundra. If the northern hemisphere tundra is divided into two sub‐biomes, the entire Fennoscandian tundra should be regarded as arctic rather than alpine, as proposed by Sonesson et al. (1975), Bliss (1981), Brown and Gibson (1983), and Olson et al. (2001). On the other hand, the major part of the Fennoscandian tundra is characterized by mild winters, high average snow depth, and abundance of late‐melting snowbeds. These alpine features and the prevalence of ericaceous dwarf shrubs in sites with moderate snow depths distinguish the Fennoscandian ericoid–graminoid tundra from the dwarf birch tundra of northern Russia and the north Fennoscandian inland. Permafrost, which is characteristic for the truly arctic tundra (Brown et al. 1997; Romanovski 2011), is in Fennoscandia restricted to the dwarf birch–dominated inland plateaus plus pockets of continental climate in the boreal zone and to vegetation‐free summit areas (Rapp 1982; Johansson et al. 2006; Harris et al. 2009; Farbrot et al. 2013). On the Scandes, permafrost is also found at very high altitudes, but always at considerable depth below the soil surface. Therefore, it has little direct effects on the vegetation. Consequently, the vegetation is not in contact with the permafrost layer on the Fennoscandian ericoid–graminoid tundra.

The contrast between the low arctic dwarf birch tundra and the Scandinavian ericoid–graminoid tundra is profound enough to advocate that these should be treated as separate sub‐biomes. The terminology introduced by Ahti et al. (1968) provides a practical solution for dealing with such altitudinal extensions of latitudinal zones, where the impacts of altitude create moderate divergences from conditions typical for the latitudinal gross counterpart. Instead of calling such altitudinal extensions of the tundra as “alpine”, the impact of altitude on ecological conditions and vegetation can be noted with the oro‐prefix. Originally, the concept “oroarctic” of Ahti et al. (1968) was meant to indicate certain bioclimatic parallelism between northern treeless heights and arctic tundra lowlands, and the term has also been used in this meaning the majority of later comparative studies (e.g., Haapasaari 1988). However, our analyses imply that the tundra biome cannot be divided into two sub‐biomes without making one of them ecologically unduly heterogeneous. This creates an objective need for a third term. The term “oroarctic” suits this role, as it implies that the vegetation has primarily arctic affinities, but also indicates that altitude has significant impacts on ecological conditions and vegetation patterns. We thus propose that the term “alpine” should be restricted to middle‐latitude mountains, and “oroarctic” would refer to those northern highlands, where altitude has significant impact on climate and vegetation patterns. Those tundra areas, which with respect to vegetation and climate are indistinguishable from nearest pieces of indisputably arctic tundra, should be regarded as integral parts of the circumpolar arctic.

Using this nomenclature, most of the Fennoscandian tundra should be referred to as *oroarctic*. This distinction probably applies circumpolarly (Fig. 1). The alpine habitats, as defined by Körner et al. (2011), are prevalent within two latitudinal belts: from 50°N to 65°N (1.8 million km^2^) and between 40°N and 30°N (0.9 million km^2^). The gap between these latitudinal prevalence belts provides a natural limit. As we did not find any vegetational or climatic differences between the rugged parts of the Scandes and the highlands with more gentle topography, we propose that the 3 million km^2^ of “missing tundra”, excluded from the arctic sub‐biome by Walker et al. (2005) and from the alpine sub‐biome by Körner et al. (2011), should be pooled with the 1.8 million km^2^ of “northern alpine tundra” of Körner et al. (2011) to form the oroarctic sub‐biome, whose total area (4.8 million km^2^) is almost as large as the area of the strictly arctic tundra of Walker et al. (2005) (see Fig. 1). Most of the remaining tundra areas (about 1 million km^2^) on middle‐ and low‐latitude mountains form the genuinely alpine sub‐biome. Alpine areas on tropical mountains (about 0.1 million km^2^) constitute the fourth sub‐biome (Nagy and Grabherr 2009).

In concordance with Körner et al. (2011), the natural boundary between the Scandinavian oroarctic ericoid–graminoid tundra and the low arctic dwarf birch tundra appeared to depend on relative rather than absolute altitudes. Dwarf birch tundra prevails on low‐altitude plateaus on the eastern (leeward) side of the Scandinavian mountain chain, which are flanked or surrounded by higher terrain and lie only slightly above the wooded areas at lower altitudes. During cold periods, the entire terrain, from valleys to heights, is thus embraced by thermal inversions (Tenow and Nilssen 1990). During mild periods, the snow precipitation generated by circulating air masses, stays largely in the surrounding higher terrain and when the skies clear up, temperatures sink rapidly, due to the high albedo of snow‐covered, treeless surfaces. This results in cold, dry, and relatively calm winter conditions. Conversely, the northern peninsulas, where the tundra extends down to the sea level but local altitudinal differences exceed 300 m, appear to be ecologically and vegetationally indistinguishable from the Scandinavian oroarctic ericoid–graminoid tundra.

## Conclusions

The vegetation and climate patterns in the areas of western Eurasia suggest that the collective arctic–alpine tundra of the northern hemisphere could be divided into three different sub‐biomes. One is the arctic tundra (5 million km^2^), characterized by cold and snow‐poor winters and frozen soils at and slightly after the snowmelt, favoring plants that tackle the drought stress due to periodically warm weather and unavailability of water. The other is the ericoid–graminoid tundra (4.8 million km^2^), with milder and snowier winters, consisting of oroarctic tundra areas and of the most oceanic sectors of the arctic proper, characterized by ericoid heaths and grassy snowbeds. The third is the alpine tundra of mid‐ and low‐latitude mountains (about 1 million km^2^), where most sites are characterized by soils, which freeze only lightly if at all. Except for exposed ridges with freezing soils, the vegetation is graminoid dominated.

These three tundra sub‐biomes are ecologically so different that pooling them one way or another results in impractically heterogeneous units. We thus agree with Walker et al. (2005) that pooling the arctic tundra with its oroarctic extensions (e.g., Olson et al. 2001) results in a unit that is so heterogeneous that its usefulness in global change studies is questionable. Similarly, referring to oroarctic sites as arctic in the context of experimental studies can be misleading. But, perhaps most strikingly, our results also imply that pooling the oroarctic sites at altitudes of a few hundred meters with truly alpine sites at altitudes of two to three thousand meters would create an even more heterogeneous biogeographic unit, especially as the high mountains are normally also more rugged than the northern highlands. The dilemma disappears if the northern hemisphere tundra is divided into three sub‐biomes, which also seem to have quite natural boundaries, at least in Europe.

Concerning the limit of the arctic tundra, we by and large agree with Walker et al. (2005), especially with respect to North America, where bulges and invaginations in the polar tree line show that the authors include in their concept of the arctic also those altitudinal extensions of the tundra, whose altitude above surrounding terrain is modest. Whether the tree line lies at or a few hundred meters above sea level is a moot point in inland areas, where the entire landscape lies at similar or higher altitudes and lowest points of the landscape are only marginally below the tree line so that altitudinal differences are too small to influence winter climate.

In Eurasia, Walker et al. (2005) diverge from this principle and interpret the polar tree line in a way that is inconsistent with our results. The polar tree line is interpreted very narrowly and inland tundra areas, which have low arctic climate and vegetation, are excluded from the arctic. We regard this as erroneous, given that the scope of all biogeographic divisions is to map areas with comparable ecological conditions. Our results support the conclusion of Oksanen and Virtanen (1995) that the southern fringes of the hemi‐low arctic zone extend like a wedge along the eastern flanks of the Scandes (Fig. 4). With respect to vegetation and winter climate, the tundra of this area is almost identical to the tundra at the mouth of Pechora (Virtanen et al. 1999), which is definitely arctic. Therefore, as also noted by Koroleva (2006), the map of Walker et al. (2005) might still need border revisions. Our approach provides macro‐scale ecological and climatic grounds for those revisions.

## Conflict of Interest

None declared.

## Supporting information


**Appendix S1** Weather stations used for the analysis of winter climate patterns.
**Appendix S2** Sources and descriptions of vegetation data material and analysis methods.
**Appendix S3** The elevation ranges of the twelve 25 × 25 km tundra sites of Fennoscandia.
