# Supplementary material for: Where do the treeless tundra areas of northern highlands fit in the global biome system: toward an ecologically natural subdivision of the tundra biome
Source: Ecol Evol. 2015 Dec 15;6(1):143–58. doi: 10.1002/ece3.1837 (PMC4716497; doi:10.1002/ece3.1837)
Supplement: Supplementary file 1 — Appendix S1 Weather stations used for the analysis of winter climate patterns. Appendix S2 Sources and descriptions of vegetation data material and analysis methods. Appendix S3 The elevation ranges of the twelve 25 × 25 km tundra sites of Fennoscandia. [file ECE3-6-143-s001.docx]

**SUPPORTING INFORMATION**

**Appendix S1** Weather stations from which climate data were obtained. Variables: P (precipitation), S (snow depth), and T (mean monthly temperatures).

| Region | Station | Elevation  m a.s.l. | Variables | Years/long-term normals |
| --- | --- | --- | --- | --- |
| Yamal Peninsula | Marresale | 17 | P,S,T | 1982-2012 |
|  | Salekhard | 16 | P,T | normals |
| Pechora Peninsula | Khodovarikha | 5 | P,S,T | 1999-2004 |
|  | Cape Svedskij | 5 | P,S,T | 2001-2004 |
|  | Narjan Mar | 12 | P,T | normals |
| Kanin Peninsula | Mys Mikulkin | 6 | P,S,T | 2001-2012 |
|  | Sonja | 49 | P,S,T | 2001-2012 |
| Kola Peninsula | Kanevka | 72 | P,S,T | 2001-2004, 2012 |
|  | Gremikha Bay | 12 | P,S,T | 2001-2004, 2012 |
| coastal Finnmark | Makkaur Fyr | 9 | P,S,T | 1980-2012 |
|  | Slettnes Fyr | 8 | P,S,T | 2009-2012 |
| interior Finnmark | Suolovuopmi (Čearro) | 377 | P,S,T | 1980-2012 |
|  | Čoavddatmohkki | 286 | P,S,T | 1982-2012 |
|  | Kautokeino | 319 | P,S,T | 1997-2012 |
|  | Šihččajavri | 382 | P,S,T | 1980-2012 |
| northern Scandes | Kilpisjärvi | 480 | P,S,T | 1982-2012 |
|  | Lyngen | 710 | S,T | 2012-2015 |
|  | Gátterjohkka/Katterjåkk | 515 | P,S,T | 1999-2012 |
|  | Rihccem/Ritsem | 491 | P,S,T | 1982-2012 |
|  | Mierkenis | 614 | P,T | 2001-2012 |
| southern Scandes | Fokstugu | 973 | P,S,T | 1982-2012 |
|  | Sognefjellhytta | 1413 | P,S,T | 1982-2012 |
|  | Midtlaeger | 1079 | P,S,T | 1982-1983,1995-2012 |
|  | Sandhaug | 1250 | T | 2009-2012 |
|  | Hovden | 780 | P,T | 1995-2012 |
| The Alps | Samedan | 1707 | P,T | 1982-2012 |
|  | Weissfluhjoch | 2540/2693 | P,S,T | 2006-2012 |
|  | Col du Grand St. Bernard | 2472 | P,T | 2000-2012 |
|  | Galzig | 2090 | P,S,T | 1982-2012 |
|  | Guetsch ob Andermatt | 2287 | P,S,T | 1982-2012 |
|  | Ischgl/Idalpe | 2319 | P,T | 1993-2012 |
|  | Naluns/Schlivera | 2400 | P,T | 2006-2012 |
|  | Paganella | 2129 | P,S,T | 1982-2012 |
|  | Patscherkofel | 2247 | P,S,T | 1982-2012 |
|  | Pilatus | 2129 | P,T | 2006-2012 |
|  | Rudolfshütte-Alpinzentrum | 2317 | P,S,T | 1982-2012 |
|  | Saentis (Säntis) | 2490 | P,T | 1982-2012 |
|  | Villacher Alpe | 2140 | P,S,T | 1982-2012 |

**Appendix S2 Sources and descriptions of vegetation data material and analysis methods**

*Middle latitude mountains*

1. Eastern Pyrenees. Alliance du Salicion herbaceae (Braun-Blanquet, 1948: Table 9), Empetreto-Vaccinietum (Braun-Blanquet, 1948: Table 30). Altitude 2100-2550 m a.s.l. Total 34 plots.

2. Rätische Alps. Salicetum herbaceae (Braun-Blanquet, 1975: obtained from Vetterli 1982: Appendix 3, sample numbers 1-24), less extreme snowbeds (Vetterli, 1982: Appendix 2 vegetation units 4a-c) and alpine meadow-like vegetation (Vetterli, 1982: Appendix 2 unit 7), and wind-exposed vegetation on base-poor soil (Vetterli, 1982: Appendix 2, unit 11a-b). Altitude 2300-2800 m a.s.l. Total 80 plots.

*Southern Scandes*

3. Sikilsdalen. Loiseleuria procumbens-Vaccinium uliginosum-Alectoria ochroleuca-sosiasjon (Nordhagen, 1943: Table 2), Cladonia-rike Betula nana-risheier (Nordhagen, 1943: Table 9), Vaccinium myrtillus-Cladonia silvatica-Dicranum fuscescens-sosiasjon (Nordhagen, 1943: Table 15: plots 1-15), Deschampsia flexuosa-Anthoxanthum-sosiasjon (Nordhagen, 1943: Table 37), Mosrik Salix herbacea-sosiasjon (Nordhagen, 1943: Table 42). Altitude 1100-1350 m a.s.l. Total 97 plots.

4. Rondane. Phyllodoco-Vaccinion myrtilli (Dahl, 1957: Table 22, 10 plots), Deschampsio-Anthoxanthio (Dahl 1957: Table 33, 10 plots). Cassiopeto-Salicion herbaceae (Dahl, 1957: Table 33, 12 plots), Loiseleurieto-Diapensietum (Dahl, 1957: Table 14, 5 plots), Cetrarietum nivalis (Dahl 1957: Table 16, 9 plots), Cladonietum alpestris (Dahl, 1957: Table 21, 10 plots). Altitude 1020–1240 m a.s.l. Total 57 plots.

5. Sylene. Sites of Nordhagen (1927) re-sampled in 1999 (Virtanen *et al.*, 2003). The vegetation units represent the following vegetation categories identified by Nordhagen (1927): Cetraria nivalis-Alectoria ochroleuca rich Loiseleuria association (15 plots), Vaccinium myrtillus heaths (35 plots), Deschampsia flexuosa -Anthoxanthum association, Cladonia gracilis variant (20 plots). Altitude 950–1050 m a.s.l. Total 70 plots.

*Northern Scandes*

6. Ivggegáisat. Oligotrophic heath vegetation types described by Oksanen & Virtanen (1995). Altitude 425–575 a.s.l. Total 56 plots.

7. Vannöya. Oligotrophic heath vegetation types described by Virtanen *et al.* (1999a). Altitude 225–375 m a.s.l . Total 56 plots.

8. Ráisduottar. Oligotrophic heath vegetation types described by Oksanen & Virtanen (1995). Altitude 500–650 m a.s.l. Total 56 plots.

*Northern Fennoscandia*

9. Dárju. Oligotrophic heath vegetation types described by Oksanen & Virtanen (1995). Altitude 550–700 m a.s.l. Total 56 plots.

10. Jávrisduottár. Oligotrophic heath vegetation types described by Oksanen & Virtanen (1995). Altitude 425–575 m, 48 plots.

11. Čearro, interior Finnmark, northern Norway. Oligotrophic heath vegetation types described by Oksanen & Virtanen (1995). Alt. c. 450 m a.s.l., Total 68 plots.

12. Cáhppisduottar, northern Finnish Lapland. Oligotrophic heath vegetation types described by Oksanen & Virtanen (1995). Alt. 350–425 m a.s.l. Total 48 plots.

13. Varanger Peninsula, Raggonjárga. Oligotrophic heath vegetation types described by Virtanen *et al.* (1999a). Alt. 25–175 m a.s.l. Total 56 plots.

*Northwestern Russia*

14. Kola Peninsula, Teriberka. Tundra heath vegetation c. 50–100 m a.s.l. 24 sample plots. H. Tømmervik (unpublished data).

15. Kola Peninsula, Kachkovsky bay. Oligotrophic heath vegetation types described by Virtanen *et al*. (1999b). Two slope gradients from the river valley to hilltops Altitude 90–130 m a.s.l. Eight transects. 64 sample plots.

16. Kanin Peninsula. Oligotrophic heath vegetation types described by Virtanen *et al*. (1999b). Two slope gradients from the river valley to hilltops 20–70 m a.s.l. Ten transects. 72 sample plots.

17. Tundra areas northwest of the mouth of the River Pechora. Oligotrophic tundra vegetation types described by Virtanen *et al*. (1999b). No slope gradient. Ten transects. Five transects on relatively level tundra, five on river bank with more pronounced topographic variation. Altitude 10–20 m a.s.l. 80 sample plots.

*Western Siberia*

18. Southern Yamal Peninsula. Tundra vegetation types on undisturbed sites. No slope gradient. Six transects. Altitude c. 50 m a.s.l. 48 sample plots. B.C. Forbes (unpublished data).

19. Middle Yamal Peninsula. Tundra vegetation types described by Virtanen *et al.* (1999b). No slope gradient. Six transects. Altitude c. 20 m a.s.l. 48 sample plots.

**References**

Braun-Blanquet J (1948) *La Végétation alpine des Pyrénées Orientales*. Monografía de la Estación de Estudios Pirenaicos, Barcelona. 306 pp.

Dahl, E. (1957) Rondane. Mountain vegetation in South Norway and its relation to the environment. *Skrifter Norske Videnskaps‑Akademi, Matematisk‑Naturvidenskapelige klasse*, **1956(3)**, 1‑374.

Forbes, B.C., Arctic Centre, University of Lapland, Finland, unpublished data from southern Yamal Peninsula, N Russia.

Nordhagen, R. (1927) Die Vegetation und Flora des Sylenegebietes. I. Die Vegetation. *Skrifter Norske Videnskaps‑Akademi Oslo, I. Matematisk‑Naturvidenskapelige klasse*, **1927(1)**, 1‑612.

Nordhagen, R. (1943) Sikilsdalen og Norges fjellbeiter. En plantesosiologisk monografi. *Bergens Museum Skrifter*, **22**, 1-607.

Tømmervik, H., Tromsø, Norway, unpublished data from northern Kola Peninsula, N Russia.

Oksanen, L. & Virtanen, R. (1995) Topographic, altitudinal and regional patterns in continental and suboceanic heath vegetation of northern Fennoscandia. *Acta Botanica Fennica*, **153**, 1-80.

Vetterli, L. (1982) Alpine Rasengesellschaften auf Silikatgestein bei Davos. V*eröffentligungen Geobotanische Institut Rübel, Zürich*, **76**, 1-92.

Virtanen, R., Pöyhtäri, P. & Oksanen, L. (1999a) Topographical and altitudinal patterns of heath vegetation on Vannøya and N Varanger Peninsula, oceanic northern Norway. *Acta Botanica Fennica*, **167**, 3-28.

Virtanen, R., Oksanen, L. & Razzhivin, V.Yu. (1999b) Topographical and regional patterns in tundra heath vegetation from northern Fennoscandia to Taymyr Peninsula. *Acta Botanica Fennica*, **167**, 29-83.

Virtanen, R., Eskelinen, A. & Gaare, E. (2003) Long-term changes in alpine plant communities in Norway and Finland. *Alpine biodiversity in Europe* (ed. by L. Nagy, G. Grabherr, Ch. Körner and D.B.A. Thompson), pp. 411-422. Ecological Studies 167. Springer, Berlin.

**Appendix S3** The elevation ranges of the twelve 25 x 25 km tundra sites of Fennoscandia. The highest and lowest points of the twelve 25 x 25 km quadrats (see Figure 6) used in the analysis of abundance relationships between different types of oligotrophic tundra heaths. The approximate altitude of treeline is provided as a point of reference.

| Quadrat | Site | Lowest | Highest | Difference | Treeline |
| --- | --- | --- | --- | --- | --- |
|  |  | m above sea level | | m | m above sea level |
| 1 | Varanger | 19 | 580 | 561 | 50 |
| 2 | Spiertanjárga | 0 | 510 | 510 | 200 |
| 3 | Porsanger | 0 | 562 | 562 | 100 |
| 4 | Iešjávri | 338 | 600 | 262 | 400 |
| 5 | Jávrisduottar | 360 | 645 | 285 | 450 |
| 6 | Davvavuopmi | 472 | 805 | 333 | 600 |
| 7 | Ultevis | 370 | 1420 | 1050 | 700 |
| 8 | Padjelántta | 580 | 1815 | 1235 | 600 |
| 9 | Björkfjället | 585 | 1600 | 1015 | 750 |
| 10 | Hardangervidda N | 1055 | 1450 | 395 | 1050 |
| 11 | Hardangervidda S | 705 | 1605 | 900 | 1050 |
| 12 | Sirdalsheiane | 370 | 1298 | 928 | 900 |
